# Interoception and psychopathology: A developmental neuroscience perspective

**DOI:** 10.1016/j.dcn.2016.12.006

**Published:** 2016-12-23

**Authors:** Jennifer Murphy, Rebecca Brewer, Caroline Catmur, Geoffrey Bird

**Affiliations:** a MRC Social, Genetic and Developmental Psychiatry Centre, Institute of Psychiatry, Psychology, and Neuroscience, King’s College London, London, UK; b School of Psychology, The University of East London, London, UK; c Institute of Cognitive Neuroscience, UCL, London, UK; d Dept of Experimental Psychology, University of Oxford, Oxford, UK

**Keywords:** Interoception, Development, Psychopathology, Emotion, Insula, Alexithymia

## Abstract

Interoception refers to the perception of the physiological condition of the body, including hunger, temperature, and heart rate. There is a growing appreciation that interoception is integral to higher-order cognition. Indeed, existing research indicates an association between low interoceptive sensitivity and alexithymia (a difficulty identifying one’s own emotion), underscoring the link between bodily and emotional awareness. Despite this appreciation, the developmental trajectory of interoception across the lifespan remains under-researched, with clear gaps in our understanding. This qualitative review and opinion paper provides a brief overview of interoception, discussing its relevance for developmental psychopathology, and highlighting measurement issues, before surveying the available work on interoception across four stages of development: infancy, childhood, adolescence and late adulthood. Where gaps in the literature addressing the development of interoception exist, we draw upon the association between alexithymia and interoception, using alexithymia as a possible marker of atypical interoception. Evidence indicates that interoceptive ability varies across development, and that this variance correlates with established age-related changes in cognition and with risk periods for the development of psychopathology. We suggest a theory within which atypical interoception underlies the onset of psychopathology and risky behaviour in adolescence, and the decreased socio-emotional competence observed in late adulthood.

## Highlights

• Reviews literature on the development of interoception.
• Atypical interoception may cause onset of psychopathology in adolescence.
• Atypical interoception may also cause risky behaviour in adolescence.
• Interoceptive changes may underlie socio-emotional changes in late adulthood.

## 1 Outline

Interoception is described as the perception of the internal state of one’s body; as such, signals including those relating to hunger, temperature, heart rate, and blood sugar levels are all interoceptive in nature. These bodily signals are thought to be represented within the insula and anterior cingulate cortex (ACC), leading these structures to be collectively referred to as the ‘interoceptive cortex’ (Craig, 2002; but see Damasio et al., 2012, Feinstein et al., 2016, discussed in more detail in Section 2). Accurate perception of internal states is unsurprisingly important for their regulation, with atypical interoceptive sensitivity (Garfinkel et al., 2015a; see Table 1: Glossary) being associated with conditions such as obesity and diabetes (Herbert and Pollatos, 2014, Pauli et al., 1991). In addition to the importance of interoception for physical health, recent research has suggested that interoception may play a role in higher-order cognition, such as in emotional memory (Pollatos and Schandry, 2008), and learning and decision making (Werner et al., 2009). Despite increasing appreciation of the importance of interoception, little is known about how interoceptive ability develops, and its stability across the lifespan. As research directly examining interoception across development is scarce, the current article also draws upon research examining developmental changes in the prevalence of alexithymia. Alexithymia is a sub-clinical condition which has traditionally been defined in terms of difficulties identifying and describing one’s own emotions (Nemiah et al., 1976), but recent evidence suggests that alexithymia may be characterised by atypical interoceptive sensitivity, rather than with specific difficulties in the affective domain (Shah et al., 2016; Gaigg et al., in press; Herbert et al., 2011, Brewer et al., 2016a, Brewer et al., 2016b, Longarzo et al., 2015, Näring and Van der Staak, 1995). Accordingly, we interpret increases in the prevalence of alexithymia at certain developmental stages as likely markers of atypical interoception, but of course the association between alexithymia and atypical interoception should be examined across development. While evidence using objective measures of interoception is clearly preferable, research on alexithymia is more common than that on interoception in the developmental literature. Rather than attempting to provide a full review of the adult interoception literature, therefore, the current paper aims to combine developmental research on interoception and alexithymia, in order to present a theory of how interoception may change across development, from infancy to late adulthood, and the possible consequences of this change. Whilst we do not propose that alexithymia and impaired interoception are interchangeable terms, we do propose that where heightened rates of alexithymia are observed within a population then this should be considered a marker of atypical interoception.

**Table 1:** (Glossary) Definitions of the terminology used in this paper are provided below.

Term	Definition
***Implicit Interoceptive Perception***	A broad term referring to situations in which subconscious processing of interoceptive signals impacts on bodily states, neural activity, and ongoing cognition. This includes homeostatic regulation but also encompasses various effects such as those on perception and memory for stimuli presented at different stages of the cardiac cycle, and at differing levels of physiological arousal (see text).
***Explicit Interoceptive Perception***	Conscious representation of interoceptive signals. Note that this broad definition would include, at the lowest level, detection of the onset or change of an interoceptive signal, and discrimination of interoceptive signals and knowledge of their intensity at higher levels. Explicit interoceptive perception has been suggested to be a three dimensional construct by Garfinkel and colleagues (see text), including interoceptive sensitivity, sensibility and awareness.
***Interoceptive Sensitivity***	Accurate detection and discrimination of interoceptive signals on explicit interoception tasks such as the Heartbeat Tracking Task.
***Interoceptive Sensibility***	An individual’s self-reported interoceptive ability.
***Interoceptive Awareness***	A metacognitive measure indexing the degree to which an individual’s interoceptive sensibility accurately reflects their interoceptive sensitivity.
***Interoceptive Ability***	A ‘catch-all’ term encompassing all aspects of explicit and implicit interoception.
***Atypical Interoception***	Unusually high or low sensitivity, sensibility or awareness. Used to indicate an interoceptive profile that is not typically observed in the general population.

Section 2 of this article briefly defines interoception, outlines methods to measure interoceptive ability, and argues for the importance of understanding the development of interoception with respect to both typical cognition and psychopathology. Section 3 reviews the available literature on the development of interoception across four stages of life: infancy, childhood, adolescence and late adulthood. It is argued that cross-sectional evidence from both objective interoceptive tests and alexithymia measures indicates that interoceptive ability may decrease in adolescence and late adulthood, and that this change may underlie the emergence of psychiatric disorders and emotion recognition difficulties across these stages, respectively. Section 4 outlines conclusions from this survey of the literature and recommendations for future progress.

## 2 Interoception: characterisation, measurement, and relevance to health

It is widely agreed that interoception refers to the perception of the internal state of one’s body; such a simple definition, however, hides a great deal of uncertainty. Whilst early definitions included visceral (internal) sensations only (e.g., Craig, 2002, Fowler, 2003), the term interoception has been broadened such that the definition is frequently taken to include certain bodily signals that do not readily meet the criteria to be considered internal (e.g., sensual or affective touch and tickle) but which are all processed using the same neural pathways as interoceptive information. Thus, more recent definitions of interoception include any bodily information that is sent either via 1) small diameter (unmyelinated) C-fibres or (myelinated) Aδ-fibres, lamina I, the spinothalamic tract and then onto the insula and anterior cingulate cortex (Craig, 2002), or 2) cranial nerves (vagus and glossopharyngeal) to the nucleus of the solitary tract (Critchley and Harrison, 2013). Whilst the insula and anterior cingulate cortex are thought to be the regions where interoceptive signals converge (e.g., Craig, 2002) and are therefore crucial for interoceptive awareness, a case study of one patient with bilateral insula damage questions this proposal. Despite their insula damage, the patient’s perception of pain, response to tickling, and to some extent taste, remained relatively intact (Damasio et al., 2012). Typical pain perception following damage to both the insular and anterior cingulate cortex was also reported in a separate case study of another patient (Feinstein et al., 2016). While clearly not consistent with the proposal that intact insular and anterior cingulate cortices are necessary for interoception, interpretation of these findings is made difficult by the fact that the patients presumably experienced typical interoceptive abilities for at least 28 years prior to insula or ACC damage. Therefore, while the precise definition of interoception and the neural regions supporting interoception remain a matter of debate, for the purposes of the current article interoception is defined as the perception of any bodily state mediated by the neural pathways described above (Craig, 2002, Critchley and Harrison, 2013).

The difficulties in defining interoception are reflected in its characterisation and measurement. For example, it has been suggested that individual differences in interoceptive ability should be considered on three dimensions rather than one: objective interoceptive sensitivity (the degree to which an individual can accurately perceive the state of their body); subjective interoceptive sensibility (an individual’s beliefs about their interoceptive accuracy/sensitivity); and interoceptive awareness (a metacognitive measure which reflects the degree to which an individual’s sensibility accurately reflects their sensitivity; Garfinkel et al., 2015a; see Table 1: Glossary); with interoceptive sensitivity serving as the core construct. It is worth noting that these dimensions generally refer to explicit interoception (conscious perception of, or beliefs about, one’s internal state), but that interoception can also be implicit, for example during homeostasis, when subconscious perception of internal states allows regulation of the bodily state, or when subconscious perception of internal states alters behavioural, neural or bodily responses in the absence of conscious awareness. It is clear then that individual differences in ‘interoceptive ability’ may be a product of, 1) the interoceptive signal itself (e.g. there may be individual differences in the extent to which individuals become aroused, meaning that for some individuals there is a weaker interoceptive signal to be perceived), 2) differences in the transduction of the interoceptive signal or its transmission to the central nervous system (and there may be developmental influences on this process; Feng et al., 2013), 3) the degree to which unconscious perception of interoceptive states impacts on bodily states, neural activity, and ongoing cognition (Azevedo et al., 2016a, Martins et al., 2014, Suzuki et al., 2013, Garfinkel et al., 2013, Garfinkel et al., 2014, Fiacconi et al., 2016, Gray et al., 2009, Gray et al., 2010, Gray et al., 2012; which we refer to as ‘implicit interoception’ within this paper), or 4) the degree to which individuals can consciously perceive, and recognise/differentiate, interoceptive signals (which we refer to as ‘explicit interoception’; see Table 1 for a Glossary).

Measurement of explicit interoceptive sensitivity has relied almost exclusively on tasks assessing heartbeat perception, typically heartbeat tracking and heartbeat discrimination tasks (e.g., Schandry, 1981, Katkin et al., 1983, Whitehead et al., 1977). In the former, participants are required to count their heartbeats over a specified interval and their count is compared to the actual number of heartbeats in that period. In the latter, participants hear two auditory stimuli, one in-phase with their heartbeat and one slightly delayed, and are required to indicate which signal is in-phase. While the reliability of these tests has been well-established (e.g., Brener and Kluvitse, 1988, Jones, 1994, Wildman and Jones, 1982), several factors affect their suitability for research. First, the tests are extremely insensitive at lower ability levels; approximately 30% of typical, healthy individuals have no conscious awareness of their heartbeat at all (Khalsa et al., 2009a). This insensitivity makes them ill-suited to index interoceptive ability in populations that may be characterised by reduced interoceptive ability caused by ill health or developmental stage. Second, heartbeat may be perceived via (exteroceptive) touch receptors due to the vibration of the chest wall (Khalsa et al., 2009a, Khalsa et al., 2009b). The degree to which the heartbeat may be perceived via this route depends on factors such as the percentage of body fat (Rouse et al., 1988), systolic blood pressure (O'Brien et al., 1998) and resting heartrate and heartrate variability (Knapp-Kline and Kline, 2005). Again, all of these factors may change as a function of developmental stage (Umetani et al., 1998, St-Onge, 2005, Franklin et al., 1997, Yashin et al., 2006).

The almost exclusive use of cardiac-based measures of interoception reflects what is often an implicit assumption in the literature − that interoceptive sensitivity represents a unitary construct. It is assumed that those who exhibit a great deal of perceptual sensitivity for their heart rate will also be good at perceiving other interoceptive signals such as temperature or taste (e.g., Herbert et al., 2012). Such an assumption is surprising when we compare interoception to exteroception (the perception of the external world); one would not necessarily expect someone with good hearing to have good visual acuity, for example. In contrast to exteroceptive information, however, interoceptive information is processed within broadly the same neural areas (Craig, 2002), and this distinction may be relevant for our understanding of the structure of interoceptive ability. The handful of studies assessing the degree to which interoceptive ability in one domain is associated with ability in another domain are inconclusive. Certain studies do support the assumption of a unitary interoceptive ability to some degree: In two studies, for example, perception of one’s heartbeat correlated moderately with the perception of gastric distension (r = 0.5; Whitehead and Drescher, 1980, Herbert et al., 2012). In contrast, other studies comparing interoceptive accuracy across cardiac and respiratory domains find a poor correlation between sensitivity across these interoceptive domains (Pollatos et al., 2016, Garfinkel et al., 2016). Such fractionation of interoceptive accuracy is supported by early research indicating no relationship between participants’ ability to discern the presence or absence of high blood pressure, sweaty hands, or shortness of breath (Steptoe and Vögele, 1992). At the neural level, electrical stimulation studies also indicate a certain degree of fractionation; in a sample of 5 epileptic patients, stimulation of distinct regions of insular cortex was associated with distinct interoceptive sensations (Stephani et al., 2011). At present, therefore, the extent to which interoception can be considered a unitary phenomenon remains unclear. Importantly, the issue of whether interoception is unitary is of direct theoretical relevance. Most models of the contribution of interoception to higher-order cognition assume a unitary structure, referring to interoception as a whole, as opposed to discussing specific domains such as cardiac-interoception (e.g. Seth, 2013, Quattrocki and Friston, 2014). A unitary structure may not exist and, even if it does in some populations, may not necessarily be continuous over development, or in certain neurodevelopmental and psychiatric conditions. The explicit investigation of interoception across domains is, therefore, essential across developmental stages as well as across atypical populations.

Despite ambiguity surrounding the definition and measurement of interoception, the notion that interoception may be fundamentally important for higher order abilities has recently begun to gain traction. Interoception has been argued to underpin selfhood and self-awareness (Seth, 2013) and sociocognitive and socioaffective ability (e.g., Quattrocki and Friston, 2014). For example, Quattrocki and Friston (2014) suggest that infants associate interoceptive signals of warmth and satiety with their caregiver’s face, which in turn drives attachment behaviour and the development of endogenous social attention. In their model, aberrant interoception (a failure of interoceptive sensitivity) is theorised to prevent the contextualisation of interoceptive signals and impair associative learning between internal states and external cues. These authors suggest that this in turn results in a lack of integration between interoceptive signals and other sensory information, resulting in an impoverished sense of self (the combination of interoception and exteroceptive information is thought to be necessary in order to represent the self as a single entity, distinct from others), reduced sensory attenuation, and ultimately social difficulties.

A significant role for interoception in higher-order cognition is supported by empirical evidence demonstrating that interoceptive ability predicts competence in a variety of emotional domains as well as in learning and decision-making. Within the affective domain, interoception appears to be necessary for all aspects of emotional processing. Much of this evidence utilises an individual differences approach to demonstrate that, across individuals, interoceptive sensitivity is correlated with emotional stability (Schandry, 1981), emotion regulation (Füstös et al., 2013), and emotional intensity (the tendency to experience more extreme emotions with greater awareness and depth of experience; Füstös et al., 2013, Herbert et al., 2010, Pollatos et al., 2007a, Pollatos et al., 2007b, Wiens et al., 2000). Indeed, the vast majority of current theories of emotion suggest that both interoceptive signals and cognitive evaluation of one’s internal and external environment contribute towards emotional experience (Schachter and Singer, 1962, Critchley and Nagai, 2012, Garfinkel and Critchley, 2013, Gendron and Barrett, 2009, Seth, 2013).

Within learning and decision-making, most classic theories of learning apportion a crucial role to signals of punishment and reward (e.g. within operant conditioning), making the accurate perception and recognition of these signals fundamental to learning (e.g. Katkin and Wiens, 2001, Pollatos and Schandry, 2008, Werner et al., 2009). Equally fundamental are theories of value in decision-making − where the aim of decision-making is to select the option with the highest value. It is clear that value may be impacted by interoceptive state (the value of water is higher when dehydrated than when not), or be interoceptive in nature (as in the drive for primary reinforcers such as sex and food). Some theories ascribe a more fundamental role to interoception, by suggesting that decision-making is guided by stored representations of the bodily consequences of stimuli and responses. These stored representations, provided they can be perceived, are a further source of information when calculating the value of options (Damasio, 1994). Regardless of one’s theoretical standpoint, however, it is clear that accurate perception and recognition of interoceptive signals is necessary for learning and decision-making.

A growing body of evidence supports the relevance of interoceptive ability for risky decision-making. For example, Werner et al. (2009) found that scores on the heartbeat tracking task predicted performance on the Iowa Gambling Task, which relies on the ability to learn which of four risky options are advantageous and which are disadvantageous. Data consistent with this finding were obtained by Dunn et al. (2010), who used a modified version of the Iowa Gambling Task to demonstrate that when arousal cues favoured adaptive choices those with better interoception made better choices, yet when arousal cues favoured maladaptive choices individuals with better interoception made worse choices than individuals with poor interoception. Finally, a study by Sokol-Hessner et al. (2015) built upon previous work demonstrating that individual differences in physiological arousal are correlated with individual differences in loss aversion (the overweighting of losses with respect to equal gains) during risky decision-making. Sokol-Hessner et al. (2015) predicted that the degree to which these interoceptive physiological signals of arousal can be perceived is likely to modulate the impact of arousal on loss aversion during risky decision-making. Results confirmed their prediction, demonstrating that individuals with increased interoceptive sensitivity were more loss averse, providing further evidence of an impact of interoceptive awareness on risky decision-making.

Theories and evidence supporting the role of interoception in typical cognition highlight the potential relevance of atypical interoception for psychopathology, with ‘atypical interoception’ encompassing both atypically high or low interoceptive ability (see Table 1: Glossary). While certain conditions have long been associated with atypically low interoceptive sensitivity (such as Feeding and Eating Disorders: Pollatos et al., 2008, Klabunde et al., 2013), the consequences for other disorders are only just being realised (see Khalsa and Lapidus, 2016, Brewer et al., 2015b, Brewer et al., 2016a, Brewer et al., 2016b). For example, Quattrocki and Friston (2014) presented a mechanistic neurobiological model within a predictive coding framework to explain how an interoceptive failure may give rise to Autism Spectrum Disorder (henceforth ‘autism’). This model was challenged by Brewer et al. (2015a), who argued that alexithymia, which frequently co-occurs with autism (Berthoz and Hill 2005), not autism itself, is characterised by a general interoceptive impairment. The predictions of these competing models have been directly tested by comparing the performance of groups of autistic and typical individuals with varying degrees of alexithymia on the heartbeat tracking task described above (Shah et al., 2016), and on tests requiring interoception of arousal (Gaigg et al., in press). In each case alexithymia, not autism, was found to be associated with poor interoceptive sensitivity (Shah et al., 2016; Gaigg et al., in press), which was not accounted for by differences in the magnitude of interoceptive signals (Gaigg et al., in press). These data confirm the findings of earlier studies demonstrating poor cardiac sensitivity in typical individuals with high levels of alexithymia (Herbert et al., 2011) and higher self-reported interoceptive confusion in alexithymic individuals (Brewer et al., 2016a, Longarzo et al., 2015). The characterisation of alexithymia as a generalised impairment of interoception is also supported by research associating the anterior insula (the brain area in which interoceptive signals concerning the state of the body converge) with both interoceptive difficulties (see Ibañez et al., 2010), and alexithymia. Structural and functional atypicalities of the anterior insula have long been associated with developmental alexithymia (e.g., Silani et al., 2008, Kano et al., 2003, Reker et al., 2010, Bird et al., 2010a, Feldman-Hall et al., 2013, Borsci et al., 2009, Ihme et al., 2013, Bernhardt et al., 2014, Goerlich-Dobre et al., 2014), and a recent study also identified damage to anterior insula as a cause of alexithymia in individuals with traumatic brain injury (Hogeveen et al., 2016). Equally consistent is the observation that an increased prevalence of alexithymia is observed in individuals with a number of physical (e.g., diabetes, obesity) and psychiatric (e.g., eating disorders and depression) conditions (e.g., Topsever et al., 2006, Pinna et al., 2011, Cochrane et al., 1993, Honkalampi et al., 2000) where poor interoception is also observed (Pauli et al., 1991, Herbert and Pollatos, 2014, Pollatos et al., 2008, Pollatos et al., 2009).

Atypically high interoception has been argued to characterise both panic disorder and anxiety syndromes (Ehlers, 1993, Clark, 1999). Indeed, individuals with heightened rates of anxiety (and panic disorder) report greater awareness of bodily sensations (see Domschke et al., 2010 for a review) and show higher interoceptive sensitivity on heartbeat tracking measures than typical individuals (e.g., Eley et al., 2004, Ehlers and Breuer, 1992, Domschke et al., 2010). Whilst early theories suggested oversensitivity was a result of increased attention to bodily signals (Ehlers, 1993), more recent proposals suggest that anxiety symptoms arise from discrepancies between the individual’s actual and expected bodily state (i.e. greater prediction error; Paulus and Stein, 2006). Despite differing proposals regarding the process that drives oversensitivity to interoceptive information in anxiety disorders, the majority of theories suggest that oversensitivity, coupled with a bias to interpret bodily signals in a negative manner, contributes to both the cognitive (e.g., worry) and behavioural (e.g., avoidance) symptoms associated with these syndromes (Ehlers, 1993; Paulus, and Stein, 2006).

The theoretical and empirical work described above demonstrates the clinical relevance of atypical interoception, and suggests that alexithymia is a marker of atypical interoception. Although further evidence is required, the relationship between alexithymia and atypical interoception may hold important implications for understanding psychopathology. Despite the current classification methods used to delineate psychiatric disorders into discrete categories, recent research indicates substantial overlap between disorder categories, so much so that one factor − the ‘p-factor’^1^- appears to underlie a number of conditions that are presently assumed to be isolated entities (Caspi et al., 2014, Laceulle et al., 2015). The p-factor subsumes the previous two-factor structure of psychopathology which incorporated an internalising and externalising factor (e.g., Krueger et al., 1998) by demonstrating that both of these factors load onto a single factor. We have recently proposed that interoceptive ability constitutes the p-factor (for a more detailed discussion see Brewer et al., 2016a, Brewer et al., 2016b), based on evidence of interoceptive difficulties in a broad range of psychiatric conditions. For example, poor interoceptive sensitivity has been observed in individuals with depression (see Harshaw, 2015) and schizophrenia (Ardizzi et al., 2016). Likewise, high obsessive compulsive traits in non-clinical samples have been associated with reduced propensity to utilise internal bodily signals for gauging arousal (Lazarov et al., 2010). The contribution of atypical interoception to a number of other disorders including addiction (see Verdejo-Garcia et al., 2012, Naqvi and Bechara, 2010), eating disorders, somatic symptom disorders and obsessive compulsive disorder (see Khalsa and Lapidus, 2016, Brewer et al., 2015b, Brewer et al., 2016a, Brewer et al., 2016b, Stern, 2014) is also well-recognised. In contrast, atypically heightened interoceptive sensitivity is associated with anxiety and panic disorder (Paulus and Stein, 2006, Ehlers and Breuer, 1992). Alexithymia has been found to co-occur with a number of disorders − including all of the disorders loading onto the externalising factor described by Caspi et al. (2014), such as alcohol (Rybakowski et al., 1988) and Substance Abuse (Michael, 1990), and Conduct Disorder (Deborde et al., 2015), as well as conditions that load onto the internalising factor, such as Major Depressive Disorder (Honkalampi et al., 2000), Anxiety (Hendryx et al., 1991) Obsessive Compulsive Disorder (Grabe et al., 2006) and Schizophrenia (Van't Wout et al., 2007). Whilst a high pattern of co-occurrence is not unique to alexithymia, these findings, together with the association between alexithymia and poor interoception, are consistent with the idea that atypical interoception may be the ‘p-factor’ accounting for symptom commonalities between psychiatric disorders. Under this model atypical interoception would contribute towards the emotional difficulties (such as emotion regulation, expression, recognition, empathy, and emotional lability), learning and decision-making impairments, and sensory symptoms (including affective touch, pain, and somatic symptoms) seen across a number of diagnostic categories.

This model is supported by several studies demonstrating the impact of alexithymia on socio-cognitive ability, particularly in the affective domain, in both typical and atypical populations. A large body of research demonstrates that alexithymia impairs emotion recognition from both facial (Grynberg et al., 2012) and vocal (Heaton et al., 2012) stimuli in typical individuals, while further studies suggest it is also responsible for the atypical emotion expression, recognition, empathy, and eye-fixation seen in some individuals with autism (Bird et al., 2010a, Bird et al., 2011, Cook et al., 2013, Heaton et al., 2012, Oakley et al., 2016, Trevisan et al., 2016) and Feeding and Eating Disorders (Brewer et al., 2015b). If it is the case that alexithymia represents a general impairment in the interoceptive domain, these findings suggest that intact interoceptive abilities are crucial for successful socio-emotional functioning. Together, research on the importance of interoception for the development of typical cognition (in the emotional, learning and decision-making, sensory, and social domains) and its potential role in the aetiology and/or symptomatology of a number of psychiatric conditions, make understanding the development of interoceptive ability, and the relationship between interoception and alexithymia, across the lifespan an urgent research goal. If, as we propose, interoception does represent the ‘p-factor’, understanding why heightened and impaired interoception is associated with distinct pathologies (e.g., anxiety, depression) may shed light on the mechanism by which interoception confers risk and resilience to pathology. In addition, identifying periods of significant developmental change in interoceptive ability may explain periods of enhanced risk for the development of psychopathology, and why some individuals develop symptoms despite prior typical development.

## 3 Interoception across the lifespan

Research on the development of interoception is not plentiful, but where it exists we provide an overview below. Where research is completely lacking, such as in infancy, we outline the measures that could be used to make progress, and the issues surrounding their use. Where data are available that rely upon measures of alexithymia rather than explicit tests of interoceptive ability, we incorporate this evidence in order to provide a more comprehensive review of the existing evidence subject to the assumptions detailed in Section 2. Identifying the gaps in our understanding of the development of interoception will, we hope, lead to these gaps being addressed, and to an understanding of the causal mechanisms by which interoception contributes to the development of higher-order cognition.

### 3.1 Interoception in infancy

It is immediately apparent that research on interoception in infancy must adopt very different methods to research in older individuals. Existing tests of interoceptive sensitivity for heartbeat and arousal rely on verbal comprehension and report − participants are asked to report the number of heartbeats in a given period or their subjective degree of arousal. Such methods are not available to researchers working with pre-verbal infants, necessitating alternative methods of assessing interoceptive ability. We suggest that one possible test would be to employ an implicit interoceptive discrimination task whereby stimuli (tones/flashes) are presented either in phase or out of phase with the infant’s heartbeat or rate of respiration. Interoceptive sensitivity could then be established using visual preference methods or rate of sucking on non-nutritive pacifiers. This implicit discrimination task could then be used to test developmental models linking interoception to the development of social ability (e.g. Quattrocki and Friston, 2014) by assessing the degree to which interoceptive ability predicts orientation to social stimuli (Morton and Johnson, 1991), for example, in infancy, as well as higher-order cognition at subsequent developmental stages.

Despite a lack of explicit interoception tests suitable for use in infancy, there is evidence consistent with the notion that implicit interoception emerges early in development. For example, Fairhurst et al. (2014) noted heart rate deceleration in 9 month old infants in response to caregiver stroking when it was light and of medium velocity (∼3 cm per second), the speed at which unmyelinated C-tactile fibres responsible for the transmission of interoceptive information respond (Löken et al., 2009). Similarly, a body of research indicates that taste sensitivity, and alimentary interoception (including hunger, thirst and satiety) emerges early in development (e.g., Harshaw, 2008). Whilst such evidence indicates that implicit interoception is present early in development, the age at which explicit awareness of such interoceptive signals develops, and when interoceptive signals can be differentiated and/or recognised, remains unclear (see Harshaw, 2008).

A further question to address is whether implicit measures such as that which we propose above would measure the same ability indexed by the explicit tests of interoceptive awareness used in older individuals. It is well established in adults that presenting stimuli synchronously with the systolic phase of the cardiac cycle influences perceptual processing and memory (e.g., Azevedo et al., 2016a, Martins et al., 2014, Suzuki et al., 2013, Garfinkel et al., 2013, Garfinkel et al., 2014, Fiacconi et al., 2016, Gray et al., 2009, Gray et al., 2010, Gray et al., 2012) and that this effect is mediated by the insula (Salomon et al., 2016, Gray et al., 2009). More recent evidence also suggests a positive association between interoceptive arousal (heartrate acceleration) induced by masked emotive stimuli (e.g., a disgusted face) and metacognitive accuracy (Allen et al., 2016). These results are consistent with the suggestion that adults have an implicit awareness of at least one interoceptive signal (heartbeat), and that it impacts upon higher-order cognition. However, questions remain as to the relationship between such implicit interoceptive perception and explicit interoceptive perception. Although some studies report a correlation between explicit and implicit measures (e.g., Azevedo et al., 2016a, Garfinkel et al., 2013, Suzuki et al., 2013) others have found no association (e.g., Salomon et al., 2016, Azevedo et al., 2016b). Whilst task differences are likely to contribute to discrepant findings, it remains unclear exactly how the two are related, how explicit interoception contributes to the development of social cognition (Quattrocki and Friston, 2014), and how implicit and explicit interoception contribute to the development of the concept of the ‘self’ (Seth, 2013).

### 3.2 Interoception in childhood

Implicit interoception has been examined in children between 3 and 13 years using cortical measurement of the Heartbeat Evoked Potential (HEP; Immanuel et al., 2014). The HEP is an electrophysiological Event Related Potential occurring over somatosensory and frontal/prefrontal regions approximately 200–600 ms following the R-peak of the Electrocardiogram waveform and is thought to reflect cortical processing of cardiac information. Such activity has been observed in the absence of explicit instruction to attend to one’s heartbeat (e.g., Immanuel et al., 2014, Montoya et al., 1993, Schandry et al., 1986, Shao et al., 2011, Schulz et al., 2013) and a reduction in the magnitude of this component has been noted in the presence of another interoceptive signal (e.g., pain; Shao et al., 2011). As such, the HEP may represent implicit interoceptive perception and/or the transmission of interoceptive information independent of attentional allocation or conscious perception of one’s heartbeat (e.g., Schulz et al., 2013, Schandry et al., 1986; discussed also by Garfinkel et al., 2015b, Pollatos et al., 2016). Indeed, the magnitude of the HEP has been demonstrated to vary with explicit interoceptive sensitivity in adults (Pollatos and Schandry, 2004, Schandry et al., 1986, Katkin et al., 1991; but see Montoya et al., 1993) and can be modulated by interoceptive training (Schandry and Weitkunat, 1990). The HEP’s relationship with explicit interoceptive sensitivity (although currently only demonstrated in adulthood) means it may be a viable means to investigate interoception in younger infants as well as children, but as yet the HEP has not been investigated in infancy. Future work examining the amplitude of this component across development may shed light on the stability of implicit interoceptive perception across the lifespan.

To our knowledge the youngest age at which explicit interoception, as measured by heartbeat tracking tasks, has been examined is six years of age. While no single study has directly compared performance on the heartbeat tracking task in adults and children, data from large studies (N = 1350) in children (age range 6–11 years) indicate equivalent performance to adults (Koch and Pollatos, 2014a). The assessment of alexithymia in children is much more difficult to compare with adult data as it relies on self-report data obtained using different instruments. Comparison of adult and child data would be possible using an appropriate longitudinal design, but such data are not presently available. It is important to note, therefore, that while alexithymia measures designed for children are reliable (Rieffe et al., 2006), discrepancies arise between parent- and child-report measures (Griffin et al., 2015). The degree to which self-reported alexithymia can serve as an index of interoceptive ability in childhood is therefore currently unknown. Interestingly, improved interoceptive sensitivity, at least when measured over longer time intervals, has been observed in children with autism (Schauder et al., 2015), which is known to co-occur with alexithymia (Berthoz and Hill, 2005, Griffin et al., 2015). If this result truly reflects greater interoceptive ability, rather than increased task adherence (Shah et al., 2016), then the results are not consistent with the relationship between alexithymia and interoceptive sensitivity in adults (Herbert et al., 2011, Shah et al., 2016, Näring and Van der Staak, 1995), making investigation of this specific relationship in children a priority.

A great deal of evidence supports the predictive ability of measures of interoception for physical and mental health in childhood. Attenuated heartbeat evoked potentials (atypical implicit interoception) have been observed in children with sleep disordered breathing (Immanuel et al., 2014), while poor explicit interoceptive sensitivity has been associated with obesity and disordered eating behaviour across a 1-year period (Koch and Pollatos, 2014b). Conversely, atypically high explicit cardiac sensitivity in children has been associated with both positive and negative outcomes including increased physical activity (Georgiou et al., 2015), and panic and anxiety symptoms (Eley et al., 2004), respectively. This is in line with the positive relationship between fitness and cardiac interoception (e.g., Borg and Linderholm, 1967), and panic disorder and cardiac interoception (Van der Does et al., 2000), in adulthood. The data from both adulthood and childhood, therefore, converge to suggest that both high and low interoceptive sensitivity are associated with distinct outcomes. In addition, levels of alexithymia in childhood, as measured by child appropriate questionnaire measures (Rieffe et al., 2006), are associated with glycaemic control (Housiaux et al., 2010), with difficulty describing feelings predicting glycaemic control at follow up two years later (Housiaux et al., 2016). High levels of alexithymia are also associated with somatic symptoms (Rieffe et al., 2006) and negative mood (Nasiri et al., 2009) in children. Again, these findings are consistent with research in the adult population (Luminet et al., 2006, Taylor et al., 1992, Lundh and Simonsson-Sarnecki, 2001) and highlight the potential of examining the association between these factors and objective tests of interoception (rather than self-report measures of alexithymia) in the future.

If our proposal that atypical interoception is the common factor underlying a range of psychiatric disorders is correct, then understanding interoception and the aetiology of atypical interoception in childhood may be crucial. Evidence from twin studies indicates a substantial proportion of the variance associated with heartbeat awareness in children (Eley et al., 2007) is accounted for by non-shared environmental factors. Indeed, consistent with the proposed relationship between alexithymia and interoception, a similar heritability pattern for alexithymia has been observed in adults (Jørgensen et al., 2007). Whilst it is unclear what these non-shared environmental factors are, it is possible that for both alexithymia and interoception, childhood adversity accounts for a portion of this unexplained variance. Increased rates of psychiatric, and some physical, disorders have been associated with a number of adverse experiences in childhood including abuse, neglect (e.g., MacMillan et al., 2001, Kaplow and Widom, 2007) and poor family environment (see Repetti et al., 2002). Whilst individual differences in cardiac interoception have not yet been associated with specific childhood experiences, certain types of atypical interoception, such as altered pain perception in adulthood, have been associated with childhood abuse (e.g., Fillingim and Edwards, 2005). Further, this maltreatment is associated with structural network changes to interoceptive regions such as the insula and anterior cingulate cortex (e.g., Teicher et al., 2014). It is notable also that increased rates of alexithymia in adulthood have been associated with the many of the same adverse childhood experiences (e.g., Kench and Irwin, 2000, Berenbaum, 1996, Aust et al., 2013). Given the association between alexithymia, interoception, and adverse life experiences, examining whether these experiences, and others, represent the non-shared environmental factors that account for a proportion of individual differences across other domains of interoception is a question for future research. Furthermore, given the relationship between alexithymia and interoception, understanding alexithymia in childhood may be important in understanding the trajectory of interoception across early development, and in determining whether childhood interoceptive abilities are stable, or influenced by adversity (e.g., Martínez-Sánchez et al., 2003, Karukivi and Saarijärvi, 2014). Whilst the stability of interoceptive ability over time remains an unanswered question (De Peuter et al., 2008), longitudinal studies over an 11-year period indicate relative stability of alexithymia in adulthood (Tolmunen et al., 2011). Further studies in childhood that consider environmental influences early in development, however, are urgently required. In particular, studies assessing the environmental influences on interoceptive abilities during childhood using objective measures of interoception would shed further light on the nature of the relationship between alexithymia and interoception, and allow the relative stability of alexithymia and interoception across development to be examined.

### 3.3 Interoception in adolescence

Adolescence is the period of development following the onset of puberty characterised by many hormonal and physical changes (Coleman and Hendry, 1990, Feldman and Elliott, 1990), as well as neurological changes (see Crone et al., 2016, Blakemore et al., 2010, Blakemore and Choudhury, 2006). There is also substantial psychological change throughout adolescence; self-awareness, the capacity for strategic thought, cognitive flexibility and self-reflectiveness all develop, in addition to notable changes in identity (Blakemore and Choudhury, 2006, Rutter and Rutter, 1993). This transition from childhood to adulthood is also, however, associated with increased risk taking, accidents and criminal behaviour (e.g., Steinberg, 2007, Crone et al., 2016). Additionally, it is the period of development where many psychiatric disorders have their onset (Paus et al., 2008, Kessler et al., 2005), and is argued to have a strong impact on mental and physical health later in life. The hypothesis that atypical interoception may underpin the emergence of psychopathology would therefore be supported by evidence of atypical interoception during adolescence. Unfortunately, there is a dearth of research into interoception in adolescence using objective measures of interoceptive sensitivity. A single study reported, consistent with evidence across other stages of development, that reduced heartbeat awareness was associated with adolescent obesity, although a relatively small sample precluded observation of any meaningful effects of adolescence on heartbeat perception sensitivity itself (Mata et al., 2015).

Surprisingly, although many studies have investigated the neural correlates of interoceptive ability in adolescents with and without psychopathology, we are aware of only one study that directly contrasts neural activity evoked by interoceptive signals in adults and adolescents. May et al. (2014) compared adolescents (15–17 years), young adults (20–28 years), and mature adults (29–55 years), on subjective pleasantness, sensitivity and neural reactivity to affective touch. Whilst no differences were observed at the behavioural level, adolescents displayed greater bilateral posterior insula activation to affective touch.

Studies examining the relationship between interoception and wellbeing in adolescence suggest that atypical interoception-related neural activity is associated with poor mental and physical health. For example, in overweight adolescents, insula activation correlates positively with one’s self-reported tendency to eat in response to external cues (e.g., the sight or smell of food rather than internal signals of hunger), whilst the inverse was found for adolescents of typical weight (Mata et al., 2015). Atypical insula activation in overweight adolescents has also been associated with risky decision making, specifically the period preceding a risky choice, a pattern of results that was not observed in those of a typical weight (Delgado-Rico et al., 2013). Similarly, adolescents with substance use disorders (alcohol or cannabis) show a reduced insula response to sensual touch (a positive interoceptive signal; Migliorini et al., 2013) and an enhanced insula response to breathing load (an aversive interoceptive signal; Berk et al., 2015).

It is clear that knowledge of how the neural correlates of interoception change as a result of development, irrespective of pathology, is urgently required. A very recent study with typical female adolescents by Li et al. (2016) highlights an interesting developmental trajectory. During a gastric regulation task, in which participants were required to control their gut responses using deep breathing exercises, interoception-related activation of the dorsal anterior insula (but not posterior insula) increased linearly with age. We suggest that additional studies of the development of interoceptive ability, providing further understanding of the way in which interoceptive abilities develop in the typical population, are likely to shed light on how and why atypical interoception in adolescence might be related to the development of psychopathology.

In contrast with the lack of research explicitly investigating interoception in typical adolescence, research on alexithymia in typical adolescence is more plentiful. Large population-based studies indicate an increased prevalence of alexithymia in adolescence: whilst the prevalence of alexithymia in adults is approximately 8–10% (Linden et al., 1995, Salminen et al., 1999), rates of alexithymia between the ages of 11–18 years are approximately 18% (Gatta et al., 2014, Säkkinen et al., 2007; but see Joukamaa et al., 2007, Honkalampi et al., 2009), although the prevalence of alexithymia decreases and remains stable in late adolescence (Säkkinen et al., 2007, Gatta et al., 2014, Karukivi et al., 2014a). If an increased prevalence of alexithymia does indeed suggest atypical interoception, such a spike in adolescence, particularly in earlier adolescence, would be consistent with the idea that interoceptive impairment represents a risk factor for, or is causally associated with, the development of psychopathology during this period.

In adolescence, alexithymia is associated with a number of physical, psychiatric and social impairments, including anxiety (Karukivi et al., 2010b), delinquency (Zimmermann, 2006), depression (Honkalampi et al., 2009), social difficulties (Honkalampi et al., 2009), severe disruptive behaviour (Manninen et al., 2011), conduct disorder (Deborde et al., 2015) and eating disorder symptomatology (Karukivi et al., 2010a). In a 4 year-long longitudinal investigation, a particular aspect of alexithymia (specifically difficulty identifying one’s feelings) was found to predict the development of anxiety symptoms in late adolescence/early adulthood (Karukivi et al., 2014b). The extent to which the relationship between these factors and alexithymia is attributable to poor interoception remains an outstanding question.

Atypical interoception during adolescence may also be related to problematic cognition typically seen during this period. As highlighted previously, adolescence is a period characterised by an increased incidence of risky behaviours such as binge drinking (Kuntsche et al., 2004), risky driving (e.g., Jonah and Dawson, 1987), unprotected sex, an increased number of sexual partners (Fergus et al., 2007, Kan et al., 2010) and substance abuse (e.g., Gfroerer, 1995). Consistent with this is the fact that rates of automobile accidents (Jonah and Dawson, 1987), sexually transmitted infections (Weinstock et al., 2004) and fatal accidents (e.g., drowning; Blum and Nelson-Mmari, 2004) are significantly increased during this developmental period. The work highlighted above in Section 2 demonstrating the role of interoception in learning and decision-making, particularly risky decision-making, makes the poor interoception seen in adolescence a candidate mechanism for the increased incidence of risky behaviours in adolescence. The additional evidence for a link between interoception and all aspects of emotion processing in Section 2, also raises the possibility that atypical interoception underlies the emotional difficulties sometimes observed in this population (e.g., Thomas et al., 2007, Van der Graaff et al., 2014, Larson et al., 2002).

The available research suggests that future studies of interoception in adolescence should consider development with respect to puberty in addition to chronological age. Alexithymia appears to be stable across late adolescence (Karukivi et al., 2014b) suggesting that if interoceptive changes occur they may emerge earlier in development, and possibly follow the onset of puberty. Whilst interoception across this period of development remains unexamined, when directly compared, significant differences in the prevalence of alexithymia are observed between early and late adolescence (Säkkinen et al., 2007), with prevalence twice as high in those aged 12–13 (21.1%) in comparison to those aged 15–17 (12.7%). If, as we propose, alexithymia does indicate the presence of poor interoception, it is likely that puberty is a sensitive period for interoceptive learning as it is characterised by many bodily changes, some of which may be unexpected and undesirable (Zvolensky and Smits, 2007). If associative learning is integral in infancy for the contextualisation of interoceptive cues (Quattrocki and Friston, 2014), it is possible that during puberty, a period replete with bodily changes, associative learning may be crucial for the contextualisation of new, unexpected, visceral sensations. Atypical interoceptive learning during this period, however, may result in poor contextualisation of bodily signals and ultimately psychopathology. Thus, although infancy is typically highlighted as a key developmental period for interoception, adolescence and possibly other stages of development where bodily changes occur, such as pregnancy (and its association with post-natal depression) and the menopause (given this period’s association with higher levels of alexithymia; Ushiroyama and Sugimoto, 1993), may be equally important.

### 3.4 Interoception in late adulthood

Late adulthood is described as the period from approximately 60–65 years of age until the end of life, with the phase of 60–65 years viewed as a transitional period from middle to late adulthood (e.g., Levinson, 1978). This period of development is associated with a number of physical and cognitive changes (see Whitbourne, 2012), including interoceptive ability. Ageing is associated with decreased heartbeat awareness (Khalsa et al., 2009b), as well as increased rates of alexithymia, likely indicative of general interoceptive difficulties (Mattila et al., 2006, Joukamaa et al., 1996; but see Gunzelmann, 2002). Interoceptive sensitivity in those aged 63 and above, however, is yet to be examined with objective tests, and the lack of longitudinal data means that it remains a possibility that such evidence reflects a cohort effect rather than a true effect associated with ageing. Nevertheless, these data are consistent with the alexithymia increasing, and interoceptive ability declining, in late adulthood.

Ageing is associated with a number of other physical difficulties that are likely underpinned by, or represent, atypical interoception. These include an increased risk of dehydration (Silver, 1990), changes in temperature sensitivity (Clark and Mehl, 1971), reduced taste sensation (Stevens et al., 1995), and changes to pain thresholds and tolerance levels (see Gagliese, 2009). All of these signals are interoceptive in nature, and difficulties with their detection or recognition would lead to negative health outcomes. Whether these difficulties are caused by impaired transduction, transmission, perception, or recognition of interoceptive signals is, however, currently unclear. A reduction in a generalised ability to consciously perceive and recognise bodily signals may underlie, or at least exacerbate, physical changes which negatively impact upon the transduction or transmission of interoceptive signals (see Feng et al., 2013). The atypical interoception hypothesis may also explain the substantial individual differences among older adults within these domains. Interoceptive ability, and the degree to which it has been impacted by normal ageing, may underlie the large individual differences observed in pain sensitivity in older adults (Gagliese, 2009) and age related changes in taste sensitivity (Stevens et al., 1995), for example.

If correct, this hypothesis is likely to be of significant clinical import. For example, older adults are at a greater risk of dehydration, which is associated with adverse health outcomes and mortality (Waikar et al., 2009, Warren et al., 1994). As many as one in five older adults may be dehydrated and systematic identification of at-risk individuals is in its infancy (Fortes et al., 2015). One possibility is that older adults with atypical interoception are at a greater risk of dehydration as thirst is considered to be an interoceptive signal (see Khalsa and Lapidus, 2016). Therefore, objective tests of interoceptive ability may prove to be useful for the identification of older adults at risk of dehydration. Even quick self-report measures of interoceptive ability or alexithymia may prove to be useful screening tools for the identification of dehydration risk in the elderly.

Beyond explicit measures of interoception, as in other developmental stages, alexithymia in late adulthood has been associated with poor physical and mental health. For example, alexithymia correlates with a number of factors that confer cardiovascular risk such as body fat, blood pressure response to provocation, trait anxiety, anger, depression, perceived stress and lower levels of social support (Waldstein et al., 2002), and depression severity in those aged over 50 (Bamonti et al., 2010) and diabetes (Hintistan et al., 2013). Given the significant health risks associated with these conditions, confirmation of the role of atypical interoception (presently assumed due to links with alexithymia) is a priority for future research.

Another notable change in late adulthood is increasingly impaired emotion recognition, although significant individual differences are observed and there are mixed findings as to whether older adults have a generalised emotion recognition impairment (see Ruffman et al., 2008) or whether their difficulties are specific to certain emotions (e.g., Horning et al., 2012, West et al., 2012, Kessels et al., 2014, Isaacowitz et al., 2007, Moreno et al., 1993). A number of factors have been found to predict age related changes in emotion recognition and memory for emotional faces, including personality (Grady et al., 2007), cognitive ability (Suzuki and Akiyama, 2013, Horning et al., 2012 and processing speed (West et al., 2012). There is some evidence that rates of alexithymia might account for difficulties older adults experience with memory for faces depicting negative emotions (Grady et al., 2007), but no research to our knowledge has examined the contribution of alexithymia or interoception to emotion recognition in older adults. In younger adults, evidence suggests a relationship between poor emotion recognition and both high levels of alexithymia (Cook et al., 2013) and low levels of interoception (e.g., Terasawa et al., 2014). Given age related changes in both alexithymia (Mattila et al., 2006, Joukamaa et al., 1996; but see Gunzelmann, 2002) and interoception (Khalsa et al., 2009b) it remains possible that rates of alexithymia and/or changes in interoceptive sensitivity explain the inverted U-shaped curve in emotion recognition ability seen across development (e.g., Horning et al., 2012, Williams et al., 2009), above and beyond the other factors identified as impacting upon emotion recognition in late adulthood.

## 4 Conclusions and future directions

This article focussed on interoception across the lifespan, documenting what we currently do and do not know about how interoceptive ability changes across development. We have described current methods to define and measure interoception, and noted that the almost exclusive use of cardiac-based measures of interoceptive ability across development is problematic, particularly in the study of groups of individuals that may be characterised by atypical interoception. We also argued that interoception is important for typical higher-order cognition, particularly in the affective realm, and in learning and decision-making. We suggested that atypical interoception may represent the ‘p-factor’, a general susceptibility to psychopathology. After providing this context, we reviewed data on the development of interoception and its impact on physical and mental health across four stages of development: infancy, childhood, adolescence and late adulthood. In the absence of available data explicitly concerning interoception, we drew upon the association between alexithymia and interoception to present a theory of how changes in interoceptive ability may underpin psychological changes from childhood to late adulthood. This review revealed important foci for future research, presented here in no particular order. The first refers to the association between interoception and alexithymia, and the extent to which alexithymia can be considered a proxy for interoceptive ability across interoceptive domains and developmental stages. The second relates to the emergence and development of interoception in infancy, and the relationship between interoceptive and social abilities in this age group. The third focus for future research should be to establish how childhood adversity influences interoception, and whether adversity accounts for the non-shared environmental factors identified by twin studies of interoceptive ability. Fourth, it is crucial to examine puberty-related changes in interoception, and whether these changes predict the onset of psychiatric disorders, risky behaviour, and social difficulties in adolescence. Such research will also bear on the question of whether interoception is the p-factor underlying susceptibility to psychopathology. Fifth, whether interoceptive ability underlies age-related changes in emotional awareness in late adulthood should be investigated, and whether simple measures of interoceptive ability can be used as clinical screening tools for diabetes, dehydration, and other interoception-related difficulties in this developmental stage. Finally, a vital initial step in all this work is the development of reliable methods to examine interoception across the lifespan in order to facilitate longitudinal developmental studies.

## Funding

JM was supported by a doctoral studentship from the Economic and Social Research Council [1599941; ES/J500057/1]. GB was supported by the Baily Thomas Charitable Trust.

## Footnotes

1 The ‘p-factor’ model (Caspi et al., 2014) includes 11 disorders/symptoms: alcohol dependence, cannabis dependence, dependence on hard drugs, tobacco dependence, conduct disorder, major depression, generalized anxiety disorder, fears/phobias, obsessive-compulsive disorder, mania and positive and negative schizophrenia symptoms.

## References

[bib0005] Allen M., Frank D., Schwarzkopf D.S., Fardo F., Winston J.S., Hauser T.U., Rees G. (2016). Unexpected arousal modulates the influence of sensory noise on confidence. eLife.

[bib0010] Ardizzi M., Ambrosecchia, Buratta M.L., Ferri F., Peciccia M., Donnari S., Mazzeschi C., Gallese V. (2016). Interoception and Positive Symptoms in Schizophrenia. Hum. Neurosci..

[bib0015] Aust S., Härtwig E.A., Heuser I., Bajbouj M. (2013). The role of early emotional neglect in alexithymia. Psychol. Trauma: Theory Res. Pract. Policy.

[bib0020] Azevedo R.T., Ainley V., Tsakiris M. (2016). Cardio-visual integration modulates the subjective perception of affectively neutral stimuli. Int. J. Psychophysiol..

[bib0025] Azevedo R.T., Aglioti S.M., Lenggenhager B. (2016). Participants’ above-chance recognition of own-heart sound combined with poor metacognitive awareness suggests implicit knowledge of own heart cardiodynamics. Sci. Rep..

[bib0030] Bamonti P.M., Heisel M.J., Topciu R.A., Franus N., Talbot N.L., Duberstein P.R. (2010). Association of alexithymia and depression symptom severity in adults aged 50 years and older. Am. J. Geriatr. Psychiatry.

[bib0035] Berenbaum H. (1996). Childhood abuse, alexithymia and personality disorder. J. Psychosom. Res..

[bib0040] Berk L., Stewart J.L., May A.C., Wiers R.W., Davenport P.W., Paulus M.P., Tapert S.F. (2015). Under pressure: adolescent substance users show exaggerated neural processing of aversive interoceptive stimuli. Addiction.

[bib0045] Bernhardt B.C., Valk S.L., Silani G., Bird G., Frith U., Singer T. (2014). Selective disruption of sociocognitive structural brain networks in autism and alexithymia. Cereb. Cortex.

[bib0050] Berthoz S., Hill E.L. (2005). The validity of using self-reports to assess emotion regulation abilities in adults with autism spectrum disorder. Eur. Psychiatry.

[bib0055] Bird G., Silani G., Brindley R., White S., Frith U., Singer T. (2010). Empathic brain responses in insula are modulated by levels of alexithymia but not autism. Brain.

[bib0060] Bird G., Press C., Richardson D.C. (2011). The role of alexithymia in reduced eye-fixation in autism spectrum conditions. J. Autism Dev. Disord..

[bib0065] Blakemore S.J., Choudhury S. (2006). Development of the adolescent brain: implications for executive function and social cognition. J. Child Psychol. Psychiatry.

[bib0070] Blakemore S.J., Burnett S., Dahl R.E. (2010). The role of puberty in the developing adolescent brain. Hum. Brain Mapp..

[bib0075] Blum R.W., Nelson-Mmari K. (2004). The health of young people in a global context. J. Adolesc. Health.

[bib0080] Borg G., Linderholm H. (1967). Perceived exertion and pulse rate during graded exercise in various age groups. Acta Med. Scand..

[bib0085] Borsci G., Boccardi M., Rossi R., Rossi G., Perez J., Bonetti M., Frisoni G.B. (2009). Alexithymia in healthy women: a brain morphology study. J. Affect. Disord..

[bib0090] Brener J., Kluvitse C. (1988). Heartbeat detection: judgments of the simultaneity of external stimuli and heartbeats. Psychophysiology.

[bib0095] Brewer R., Happé F., Cook R., Bird G. (2015). Commentary on Autism, oxytocin and interoception: alexithymia, not Autism Spectrum Disorders, is the consequence of interoceptive failure. Neurosci. Biobehav. Rev..

[bib0100] Brewer R., Cook R., Cardi V., Treasure J., Bird G. (2015). Emotion recognition deficits in eating disorders are explained by co-occurring alexithymia. R. Soc. Open Sci..

[bib0105] Brewer R., Cook R., Bird G. (2016). Alexithymia: a general deficit of interoception. R. Soc. Open Sci..

[bib0110] Brewer R., Cook R., Bird G., Obhi S.S., Cross E.S. (2016). Shared interoceptive representations: the case of alexithymia. Shared Representations. Cambridge Social Neuroscience Series.

[bib0115] Caspi A., Houts R.M., Belsky D.W., Goldman-Mellor S.J., Harrington H., Israel S., Meier M.H., Ramrakha S., Shalev I., Poulton R., Moffitt T.E. (2014). The p factor one general psychopathology factor in the structure of psychiatric disorders?. Clin. Psychol. Sci..

[bib0120] Clark W.C., Mehl L. (1971). Thermal pain: a sensory decision theory analysis of the effect of age and sex on d', various response criteria, and 50% pain threshold. J. Abnorm. Psychol..

[bib0125] Clark D.M. (1999). Anxiety disorders: why they persist and how to treat them. Behav. Res. Ther..

[bib0130] Cochrane C.E., Brewerton T.D., Wilson D.B., Hodges E.L. (1993). Alexithymia in the eating disorders. Int. J. Eat. Disord..

[bib0135] Coleman J.C., Hendry L. (1990). The Nature of Adolescence.

[bib0140] Cook R., Brewer R., Shah P., Bird G. (2013). Alexithymia, not autism, predicts poor recognition of emotional facial expressions. Psychol. Sci..

[bib0145] Craig A.D. (2002). How do you feel? Interoception: the sense of the physiological condition of the body. Nat. Rev. Neurosci..

[bib0150] Critchley H.D., Harrison N.A. (2013). Visceral influences on brain and behavior. Neuron.

[bib0155] Critchley H.D., Nagai Y. (2012). How emotions are shaped by bodily states. Emot. Rev..

[bib0160] Crone E.A., Duijvenvoorde A.C., Peper J.S. (2016). Annual Research Review: neural contributions to risk‐taking in adolescence–developmental changes and individual differences. J. Child Psychol. Psychiatry.

[bib0165] Damasio A., Damasio H., Tranel D. (2012). Persistence of feelings and sentience after bilateral damage of the insula. Cereb. Cortex.

[bib0170] Damasio A.R. (1994). Descartes’ Error: Emotion, Reason, and the Human Brain.

[bib0175] De Peuter S., Van Diest U., Van den Bergh O. (2008). Interoception. Stable characteristic, or person-by-situation interaction?. Adv. Psychol. Res..

[bib0180] Deborde A.S., Vanwalleghem M.S., Aitel S. (2015). Emotion regulation in adolescents with conduct disorder and controls. L'Encephale.

[bib0185] Delgado-Rico E., Soriano‐Mas C., Verdejo‐Román J.S., Río‐Valle J., Verdejo‐García A. (2013). Decreased insular and increased midbrain activations during decision‐making under risk in adolescents with excess weight. Obesity.

[bib0190] Domschke K., Stevens S., Pfleiderer B., Gerlach A.L. (2010). Interoceptive sensitivity in anxiety and anxiety disorders: an overview and integration of neurobiological findings. Clin. Psychol. Rev..

[bib0195] Dunn B.D., Galton H.C., Morgan R., Evans D., Oliver C., Meyer M., Cusack R., Lawrence A.D., Dalgleish T. (2010). 2010). Listening to your heart how interoception shapes emotion experience and intuitive decision making. Psychol. Sci..

[bib0200] Ehlers A., Breuer P. (1992). Increased cardiac awareness in panic disorder. J. Abnorm. Psychol..

[bib0205] Ehlers A. (1993). Interoception and panic disorder. Adv. Behav. Res. Ther..

[bib0210] Eley T.C., Stirling L., Ehlers A., Gregory A.M., Clark D.M. (2004). Heart-beat perception, panic/somatic symptoms and anxiety sensitivity in children. Behav. Res. Ther..

[bib0215] Eley T.C., Gregory A.M., Clark D.M., Ehlers A. (2007). Feeling anxious: a twin study of panic/somatic ratings, anxiety sensitivity and heartbeat perception in children. J. Child Psychol. Psychiatry.

[bib0220] Füstös J., Gramann K., Herbert B.M., Pollatos O. (2013). On the embodiment of emotion regulation: interoceptive awareness facilitates reappraisal. Soc. Cogn. Affect. Neurosci..

[bib0225] Fairhurst M.T., Löken L., Grossmann T. (2014). Physiological and behavioral responses reveal 9-month-old infants’ sensitivity to pleasant touch. Psychol. Sci..

[bib0230] Feinstein J.S., Khalsa S.S., Salomons T.V., Prkachin K.M., Frey-Law L.A., Lee J.E., Tranel D., Rudrauf, D (2016). Preserved emotional awareness of pain in a patient with extensive bilateral damage to the insula, anterior cingulate, and amygdala. Brain Struct. Funct..

[bib0235] Feldman S.S., Elliott G.R. (1990). At the Threshold: The Developing Adolescent.

[bib0240] Feldman-Hall O., Dalgleish T., Mobbs D. (2013). Alexithymia decreases altruism in real social decisions. Cortex.

[bib0245] Feng P., Huang L., Wang H. (2013). Taste bud homeostasis in health, disease, and aging. Chem. Senses.

[bib0250] Fergus S., Zimmerman M.A., Caldwell C.H. (2007). Growth trajectories of sexual risk behavior in adolescence and young adulthood. Am. J. Public Health.

[bib0255] Fiacconi C.M., Peter E.L., Owais S., Köhler S. (2016). Knowing by heart: visceral feedback shapes recognition memory judgments. J. Exp. Psychol. Gen..

[bib0260] Fillingim R.B., Edwards R.R. (2005). Is self-reported childhood abuse history associated with pain perception among healthy young women and men?. Clin. J. Pain.

[bib0265] Fortes M.B., Owen J.A., Raymond-Barker P., Bishop C., Elghenzai S., Oliver S.J., Walsh N.P. (2015). Is this elderly patient dehydrated? Diagnostic accuracy of hydration assessment using physical signs, urine, and saliva markers. J. Am. Med. Dir. Assoc..

[bib0270] Fowler C.J. (2003). Visceral sensory neuroscience: interoception. Brain.

[bib0275] Franklin S.S., Gustin W., Wong N.D., Larson M.G., Weber M.A., Kannel W.B., Levy D. (1997). Hemodynamic patterns of age-related changes in blood pressure The Framingham Heart Study. Circulation.

[bib0280] Gagliese L. (2009). Pain and aging: the emergence of a new subfield of pain research. J. Pain.

[bib0285] Gaigg, S.B., Maurice, A.S.F., & Bird, G. (in press) The psychophysiological mechanisms of alexithymia in autism spectrum disorder. 10.1177/1362361316667062 27811193

[bib0290] Garfinkel S.N., Critchley H.D. (2013). Interoception, emotion and brain: new insights link internal physiology to social behaviour. Commentary on: anterior insular cortex mediates bodily sensibility and social anxiety by Terasawa et al. (2012). Soc. Cogn. Affect. Neurosci..

[bib0295] Garfinkel S.N., Barrett A.B., Minati L., Dolan R.J., Seth A.K., Critchley H.D. (2013). What the heart forgets: cardiac timing influences memory for words and is modulated by metacognition and interoceptive sensitivity. Psychophysiology.

[bib0300] Garfinkel S.N., Minati L., Gray M.A., Seth A.K., Dolan R.J., Critchley H.D. (2014). Fear from the heart: sensitivity to fear stimuli depends on individual heartbeats. J. Neurosci..

[bib0305] Garfinkel S.N., Seth A.K., Barrett A.B., Suzuki K., Critchley H.D. (2015). Knowing your own heart: distinguishing interoceptive accuracy from interoceptive awareness. Biol. Psychol..

[bib0310] Garfinkel S.N., Eccles J.A., Critchley H.D. (2015). The Heart, the brain, and the regulation of emotion. JAMA Psychiatry.

[bib0315] Garfinkel S.N., Manassei M.F., Hamilton-Fletcher G., In den Bosch Y., Critchley H., D, Engels M. (2016). Interoceptive dimensions across cardiac and respiratory axes. Philos. Trans. R. Soc. B.

[bib0320] Gatta M., Facca I., Colombo E., Svanellini L., Montagnese S., Schiff S. (2014). Alexithymia, psychopathology and alcohol misuse in adolescence: a population based study on 3556 teenagers. Neurosci. Med..

[bib0325] Gendron M., Barrett L.F. (2009). Reconstructing the past: a century of ideas about emotion in psychology. Emot. Rev..

[bib0330] Georgiou E., Matthias E., Kobel S., Kettner S., Dreyhaupt J., Steinacker J.M., Pollatos O. (2015). Interaction of physical activity and interoception in children. Front. Psychol..

[bib0335] Gfroerer J. (1995). National Household Survey of Drug Abuse (Advance Report No. 10).

[bib0340] Goerlich-Dobre K.S., Bruce L., Martens S., Aleman A., Hooker C.I. (2014). Distinct associations of insula and cingulate volume with the cognitive and affective dimensions of alexithymia. Neuropsychologia.

[bib0345] Grabe H.J., Ruhrmann S., Ettelt S., Müller A., Buhtz F., Hochrein A., Schulze-Rauschenbach S., Meyer K., Kraft S., Reck C., Pukrop R., Klosterkötter J., Falkai P., Maier W., Wagner M., John U., Freyberger H.J. (2006). Alexithymia in obsessive-compulsive disorder?results from a family study. Psychother. Psychosom..

[bib0350] Grady C.L., Hongwanishkul D., Keightley M., Lee W., Hasher L. (2007). The effect of age on memory for emotional faces. Neuropsychology.

[bib0355] Gray M.A., Rylander K., Harrison N.A., Wallin B.G., Critchley H.D. (2009). Following one's heart: cardiac rhythms gate central initiation of sympathetic reflexes. J. Neurosci..

[bib0360] Gray M.A., Minati L., Paoletti G., Critchley H.D. (2010). Baroreceptor activation attenuates attentional effects on pain-evoked potentials. PAIN^®^.

[bib0365] Gray M.A., Beacher F.D., Minati L., Nagai Y., Kemp A.H., Harrison N.A., Critchley H.D. (2012). Emotional appraisal is influenced by cardiac afferent information. Emotion.

[bib0370] Griffin C., Lombardo M.V., Auyeung B. (2015). Alexithymia in children with and without autism spectrum disorders. Autism Res..

[bib0375] Grynberg D., Chang B., Corneille O., Maurage P., Vermeulen N., Berthoz S., Luminet O. (2012). Alexithymia and the processing of emotional facial expressions (EFEs): systematic review, unanswered questions and further perspectives. PLoS One.

[bib0380] Gunzelmann T. (2002). Alexithymia in the elderly general population. Compr. Psychiatry.

[bib0385] Harshaw C. (2015). Interoceptive dysfunction: Toward an integrated framework for understanding somatic and affective disturbance in depression. Psychol. Bull..

[bib0390] Harshaw C. (2008). Alimentary epigenetics: a developmental psychobiological systems view of the perception of hunger, thirst and satiety. Dev. Rev..

[bib0395] Heaton P., Reichenbacher L., Sauter D., Allen R., Scott S., Hill E. (2012). Measuring the effects of alexithymia on perception of emotional vocalizations in autistic spectrum disorder and typical development. Psychol. Med..

[bib0400] Hendryx M.S., Haviland M.G., Shaw D.G. (1991). Dimensions of alexithymia and their relationships to anxiety and depression. J. Pers. Assess..

[bib0405] Herbert B.M., Pollatos O. (2014). Attenuated interoceptive sensitivity in overweight and obese individuals. Eat. Behav..

[bib0410] Herbert B.M., Pollatos O., Flor H., Enck P., Schandry R. (2010). Cardiac awareness and autonomic cardiac reactivity during emotional picture viewing and mental stress. Psychophysiology.

[bib0415] Herbert B.M., Herbert C., Pollatos O. (2011). On the relationship between interoceptive awareness and alexithymia: is interoceptive awareness related to emotional awareness?. J. Pers..

[bib0420] Herbert B.M., Muth E.R., Pollatos O., Herbert C. (2012). Interoception across modalities: on the relationship between cardiac awareness and the sensitivity for gastric functions. PLoS One.

[bib0425] Hintistan S., Cilingir D., Birinci N. (2013). Alexithymia among elderly patients with diabetes. Pak. J. Med. Sci..

[bib0430] Hogeveen J., Bird G., Chau A., Krueger F., Grafman J. (2016). Acquired alexithymia following damage to the anterior insula. Neuropsychologia.

[bib0435] Honkalampi K., Hintikka J., Tanskanen A., Lehtonen J., Viinamäki H. (2000). Depression is strongly associated with alexithymia in the general population. J. Psychosom. Res..

[bib0440] Honkalampi K., Tolmunen T., Hintikka J., Rissanen M.L., Kylmä J., Laukkanen E. (2009). The prevalence of alexithymia and its relationship with Youth Self-Report problem scales among Finnish adolescents. Compr. Psychiatry.

[bib0445] Horning S.M., Cornwell R.E., Davis H.P. (2012). The recognition of facial expressions: an investigation of the influence of age and cognition. Aging Neuropsychol. Cogn..

[bib0450] Housiaux M., Luminet O., Van Broeck N., Dorchy H. (2010). Alexithymia is associated with glycaemic control of children with type 1 diabetes. Diab. Metab..

[bib0455] Housiaux M., Luminet O., Dorchy H. (2016). Difficulties describing feelings to others still predicts glycaemic control up to 24 months later in children with type 1 diabetes. Diab. Metab..

[bib0460] Ibañez A., Gleichgerrcht E., Manes F. (2010). Clinical effects of insular damage in humans. Brain Struct. Funct..

[bib0465] Ihme K., Dannlowski U., Lichev V., Stuhrmann A., Grotegerd D., Rosenberg N., Kugel H., Heindel W., Arolt V., Kersting A., Suslow T. (2013). Alexithymia is related to differences in grey matter volume: a voxel-based morphometry study. Brain Res..

[bib0470] Immanuel S.A., Pamula Y., Kohler M., Martin J., Kennedy D., Nalivaiko E., Saint D.A., Baumert M. (2014). Heartbeat evoked potentials during sleep and daytime behavior in children with sleep-disordered breathing. Am. J. Respir. Crit. Care Med..

[bib0475] Isaacowitz D.M., Löckenhoff C.E., Lane R.D., Wright R., Sechrest L., Riedel R., Costa P.T. (2007). Age differences in recognition of emotion in lexical stimuli and facial expressions. Psychol. Aging.

[bib0480] Jørgensen M.M., Zachariae R., Skytthe A., Kyvik K. (2007). Genetic and environmental factors in alexithymia: a population-based study of 8,785 Danish twin pairs. Psychother. Psychosom..

[bib0485] Jonah B.A., Dawson N.E. (1987). Youth and risk: age differences in risky driving, risk perception, and risk utility. Alcohol Drugs Driv..

[bib0490] Jones G.E., Jennings J.R., Ackles P.K. (1994). Perception of visceral sensations: a review of recent findings, methodologies, and future directions.

[bib0495] Joukamaa M., Saarijärvi S., Muuriaisniemi M.L., Salokangas R.K. (1996). Alexithymia in a normal elderly population. Compr. Psychiatry.

[bib0500] Joukamaa M., Taanila A., Miettunen J., Karvonen J.T., Koskinen M., Veijola J. (2007). Epidemiology of alexithymia among adolescents. J. Psychosom. Res..

[bib0505] Kan M.L., Cheng Y.H.A., Landale N.S., McHale S.M. (2010). Longitudinal predictors of change in number of sexual partners across adolescence and early adulthood. J. Adolesc. Health.

[bib0510] Kano M., Fukudo S., Gyoba J., Kamachi M., Tagawa M., Mochizuki H., Itoh M., Hongo M., Yanai K. (2003). Specific brain processing of facial expressions in people with alexithymia: an H215O-PET study. Brain.

[bib0515] Kaplow J.B., Widom C.S. (2007). Age of onset of child maltreatment predicts long-term mental health outcomes. J. Abnorm. Psychol..

[bib0520] Karukivi M., Saarijärvi S. (2014). Development of alexithymic personality features. World J. Psychiatry.

[bib0525] Karukivi M., Hautala L., Korpelainen J., Haapasalo-Pesu K.M., Liuksila P.R., Joukamaa M., Saarijärvi S. (2010). Alexithymia and eating disorder symptoms in adolescents. Eat. Disord..

[bib0530] Karukivi M., Hautala L., Kaleva O., Haapasalo-Pesu K.M., Liuksila P.R., Joukamaa M., Saarijärvi S. (2010). Alexithymia is associated with anxiety among adolescents. J. Affect. Disord..

[bib0535] Karukivi M., Pölönen T., Vahlberg T., Saikkonen S., Saarijärvi S. (2014). Stability of alexithymia in late adolescence: results of a 4-year follow-up study. Psychiatry Res..

[bib0540] Karukivi M., Vahlberg T., Pölönen T., Filppu T., Saarijärvi S. (2014). Does alexithymia expose to mental disorder symptoms in late adolescence? A 4-year follow-up study. Gen. Hosp. Psychiatry.

[bib0545] Katkin E.S., Wiens S., Öhman A. (2001). Nonconscious fear conditioning, visceral perception, and the development of gut feelings. Psychol. Sci..

[bib0550] Katkin E.S., Reed S.D., Deroo C. (1983). A methodological analysis of 3 techniques for the assessment of individual-differences in heartbeat detection. Psychophysiology.

[bib0555] Katkin E.S., Cestaro V.L., Weitkunat R. (1991). Individual differences in cortical evoked potentials as a function of heartbeat detection ability. Int. J. Neurosci..

[bib0560] Kench S., Irwin H.J. (2000). Alexithymia and childhood family environment. J. Clin. Psychol..

[bib0565] Kessels R.P., Montagne B., Hendriks A.W., Perrett D.I., Haan E.H. (2014). Assessment of perception of morphed facial expressions using the Emotion Recognition Task: normative data from healthy participants aged 8–75. J. Neuropsychol..

[bib0570] Kessler R.C., Berglund P., Demler O., Jin R., Merikangas K.R., Walters E.E. (2005). Lifetime prevalence and age-of-onset distributions of DSM-IV disorders in the National Comorbidity Survey Replication. Arch. Gen. Psychiatry.

[bib0575] Khalsa S.S., Lapidus R.C. (2016). Can interoception improve the pragmatic search for biomarkers in psychiatry?. Front. Psychiatry.

[bib0580] Khalsa S.S., Rudrauf D., Sandesara C., Olshansky B., Tranel D. (2009). Bolus isoproterenol infusions provide a reliable method for assessing interoceptive awareness. Int. J. Psychophysiol..

[bib0585] Khalsa S.S., Rudrauf D., Tranel D. (2009). Interoceptive awareness declines with age. Psychophysiology.

[bib0590] Klabunde M., Acheson D.T., Boutelle K.N., Matthews S.C., Kaye W.H. (2013). Interoceptive sensitivity deficits in women recovered from bulimia nervosa. Eat. Behav..

[bib0595] Knapp-Kline K., Kline J.P. (2005). Heart rate, heart rate variability, and heartbeat detection with the method of constant stimuli: slow and steady wins the race. Biol. Psychol..

[bib0600] Koch A., Pollatos O. (2014). Cardiac sensitivity in children: sex differences and its relationship to parameters of emotional processing. Psychophysiology.

[bib0605] Koch A., Pollatos O. (2014). Interoceptive sensitivity, body weight and eating behavior in children: a prospective study. Front. Psychol..

[bib0610] Krueger R.F., Caspi A., Moffitt T.E., Silva P.A. (1998). The structure and stability of common mental disorders (DSM-III-R): a longitudinal-epidemiological study. J. Abnorm. Psychol..

[bib0615] Kuntsche E., Rehm J., Gmel G. (2004). Characteristics of binge drinkers in Europe. Social Sci. Med..

[bib0620] Löken L.S., Wessberg J., McGlone F., Olausson H. (2009). Coding of pleasant touch by unmyelinated afferents in humans. Nat. Neurosci..

[bib0625] Laceulle O.M., Vollebergh W.A.M., Ormel J. (2015). The structure of psychopathology in adolescence: replication of a general psychopathology factor in the TRAILS study. Clin. Psychol. Sci..

[bib0630] Larson R.W., Moneta G., Richards M.H., Wilson S. (2002). Continuity, stability, and change in daily emotional experience across adolescence. Child Dev..

[bib0635] Lazarov A., Dar R., Oded Y., Liberman N. (2010). Are obsessive–compulsive tendencies related to reliance on external proxies for internal states? Evidence from biofeedback-aided relaxation studies. Behav. Res. Ther..

[bib0640] Levinson D.J. (1978). Eras: the anatomy of the life cycle. Psychiatric Opin..

[bib0645] Li D., Zucker N.L., Kragel P.A., Covington V.E., LaBar K.S. (2016). Adolescent development of insula‐dependent interoceptive regulation. Dev. Sci..

[bib0650] Linden W., Wen F., Paulus D.L., Butcher J., Spielberger C. (1995). Measuring alexithymia: reliability, validity, and prevalence. Advances in Personality Assessment.

[bib0655] Longarzo M., D'Olimpio F., Chiavazzo A., Santangelo G., Trojano L., Grossi D. (2015). The relationships between interoception and alexithymic trait. The Self-Awareness Questionnaire in healthy subjects. Fronti. Psychol..

[bib0660] Luminet O., De Timary P., Buysschaert M., Luts A. (2006). The role of alexithymia factors in glucose control of persons with type 1 diabetes: a pilot study. Diab. Metab..

[bib0665] Lundh L., Simonsson-Sarnecki M. (2001). Alexithymia: emotion and somatic complaints. J. Pers..

[bib0670] MacMillan H.L., Fleming J.E., Streiner D.L., Lin E., Boyle M.H., Jamieson E., Duku E.K., Walsh C.A., Wong M.Y.Y., Beardslee W.R. (2001). Childhood abuse and lifetime psychopathology in a community sample. Am. J. Psychiatry.

[bib0675] Manninen M., Therman S., Suvisaari J., Ebeling H., Moilanen I., Huttunen M., Joukamaa M. (2011). Alexithymia is common among adolescents with severe disruptive behavior. J. Nerv. Mental Dis..

[bib0680] Martínez-Sánchez F., Ato-García M., Ortiz-Soria B. (2003). Alexithymia—state or trait?. Span. J. Psychol..

[bib0685] Martins A.Q., McIntyre D., Ring C. (2014). Effects of baroreceptor stimulation on performance of the Sternberg short-term memory task: a cardiac cycle time study. Biol. Psychol..

[bib0690] Mata F., Verdejo-Roman J., Soriano-Mas C., Verdejo-Garcia A. (2015). Insula tuning towards external eating versus interoceptive input in adolescents with overweight and obesity. Appetite.

[bib0695] Mattila A.K., Salminen J.K., Nummi T., Joukamaa M. (2006). Age is strongly associated with alexithymia in the general population. J. Psychosom. Res..

[bib0700] May A.C., Stewart J.L., Paulus M.P., Tapert S.F. (2014). The effect of age on neural processing of pleasant soft touch stimuli. Front. Behav. Neurosci..

[bib0705] Michael R. (1990). A preliminary investigation of alexithymia in men with psychoactive substance dependence. Am. J. Psychiatry.

[bib0710] Migliorini R., Stewart J.L., May A.C., Tapert S.F., Paulus M.P. (2013). What do you feel? Adolescent drug and alcohol users show altered brain response to pleasant interoceptive stimuli. Drug Alcohol Depend..

[bib0715] Montoya P., Schandry R., Müller A. (1993). Heartbeat evoked potentials (HEP): topography and influence of cardiac awareness and focus of attention. Electroencephalogr. Clin. Neurophysiol./Evok. Potent. Sect..

[bib0720] Moreno C., Borod J.C., Welkowitz J., Alpert M. (1993). The perception of facial emotion across the adult life span. Dev. Neuropsychol..

[bib0725] Morton J., Johnson M.H. (1991). CONSPEC and CONLERN: a two-process theory of infant face recognition. Psychol. Rev..

[bib0730] Näring G.W.B., Van der Staak C.P.F. (1995). Perception of heart rate and blood pressure: the role of alexithymia and anxiety. Psychother. Psychosom..

[bib0735] Naqvi N.H., Bechara A. (2010). The insula and drug addiction: an interoceptive view of pleasure, urges, and decision-making. Brain Struct. Funct..

[bib0740] Nasiri H., Latifian M., Rieffe C. (2009). Alexithymia and its relationship with physical complaints and emotional competency in children and adolescents. Iran. J. Psychiatry Clin. Psychol..

[bib0745] Nemiah J.C., Freyberger H., Sifneos P.E. (1976). Alexithymia: a view of the psychosomatic process. Modern Tends Psychosom. Med..

[bib0750] O'Brien W.H., Reid G.J., Jones K.R. (1998). Differences in heartbeat awareness among males with higher and lower levels of systolic blood pressure. Int. J. Psychophysiol..

[bib0755] Oakley B.F., Brewer R., Bird G., Catmur C. (2016). Theory of mind is not theory of emotion: a cautionary note on the Reading the Mind in the Eyes Test. J. Abnorm. Psychol..

[bib0760] Pauli P., Hartl L., Marquardt C., Stalmann H., Strian F. (1991). Heartbeat and arrhythmia perception in diabetic autonomic neuropathy. Psychol. Med..

[bib0765] Paulus M.P., Stein M.B. (2006). An insular view of anxiety. Biol. Psychiatry.

[bib0770] Paus T., Keshavan M., Giedd J.N. (2008). Why do many psychiatric disorders emerge during adolescence?. Nat. Rev. Neurosci..

[bib0775] Pinna F., Lai L., Pirarba S., Orru W., Velluzzi F., Loviselli A., Carpiniello B. (2011). Obesity, alexithymia and psychopathology: a case-control study Eating and Weight Disorders-Studies on Anorexia. Bulimia Obes..

[bib0780] Pollatos O., Schandry R. (2004). Accuracy of heartbeat perception is reflected in the amplitude of the heartbeat‐evoked brain potential. Psychophysiology.

[bib0785] Pollatos O., Schandry R. (2008). Emotional processing and emotional memory are modulated by interoceptive awareness. Cogn. Emot..

[bib0790] Pollatos O., Herbert B.M., Matthias E., Schandry R. (2007). Heart rate response after emotional picture presentation is modulated by interoceptive awareness. Int. J. Psychophysiol..

[bib0795] Pollatos O., Traut-Mattausch E., Schroeder H., Schandry R. (2007). Interoceptive awareness mediates the relationship between anxiety and the intensity of unpleasant feelings. J. Anxiety Disord..

[bib0800] Pollatos O., Kurz A.L., Albrecht J., Schreder T., Kleemann A.M., Schöpf V., Kopietz R., Wiesmann M., Schandry R. (2008). Reduced perception of bodily signals in anorexia nervosa. Eat. Behav..

[bib0805] Pollatos O., Traut-Mattausch E., Schandry R. (2009). Differential effects of anxiety and depression on interoceptive accuracy. Depress. Anxiety.

[bib0810] Pollatos O., Herbert B.M., Mai S., Kammer T. (2016). Changes in interoceptive processes following brain stimulation. Philos. Trans. R. Soc. B.

[bib0815] Quattrocki E., Friston K. (2014). Autism: oxytocin and interoception. Neurosc. Biobehav. Rev..

[bib0820] Reker M., Ohrmann P., Rauch A.V., Kugel H., Bauer J., Dannlowski U., Arolt V., Heindel W., Suslow T. (2010). Individual differences in alexithymia and brain response to masked emotion faces. Cortex.

[bib0825] Repetti R.L., Taylor S.E., Seeman T.E. (2002). Risky families: family social environments and the mental and physical health of offspring. Psychol. Bull..

[bib0830] Rieffe C., Oosterveld P., Terwogt M.M. (2006). An alexithymia questionnaire for children: factorial and concurrent validation results. Person. Ind. Diff..

[bib0835] Rouse C.H., Jones G.E., Jones K.R. (1988). The effect of body composition and gender on cardiac awareness. Psychophysiology.

[bib0840] Ruffman T., Henry J.D., Livingstone V., Phillips L.H. (2008). A meta-analytic review of emotion recognition and aging: implications for neuropsychological models of aging. Neurosci. Biobehav. Rev..

[bib0845] Rutter M., Rutter M. (1993). Developing Minds.

[bib0850] Rybakowski J., Ziółkowski M., Zasadzka T., Brzeziński R. (1988). High prevalence of alexithymia in male patients with alcohol dependence. Drug Alcohol Depend..

[bib0855] Säkkinen P., Kaltiala-Heino R., Ranta K., Haataja R., Joukamaa M. (2007). Psychometric properties of the 20-item Toronto Alexithymia Scale and prevalence of alexithymia in a Finnish adolescent population. Psychosomatics.

[bib0860] Salminen J.K., Saarijärvi S., Äärelä E., Toikka T., Kauhanen J. (1999). Prevalance of alexithymia and its association with sociodemographic variables in the general population of Finland. J. Psychosom. Res..

[bib0865] Salomon R., Ronchi R., Dönz J., Bello-Ruiz J., Herbelin B., Martet R., Faivre N., Schaller K., Blanke O. (2016). The Insula Mediates Access to Awareness of Visual Stimuli Presented Synchronously to the Heartbeat. J. Neurosci.

[bib0870] Schachter S., Singer J. (1962). Cognitive, social, and physiological determinants of emotional state. Psychol. Rev..

[bib0875] Schandry R., Weitkunat R. (1990). Enhancement of heartbeat-related brain potentials through cardiac awareness training. Int. J. Neurosci..

[bib0880] Schandry R., Sparrer B., Weitkunat R. (1986). From the heart to the brain: a study of heartbeat contingent scalp potentials. Int. J. Neurosci..

[bib0885] Schandry R. (1981). Heart beat perception and emotional experience. Psychophysiology.

[bib0890] Schauder K.B., Mash L.E., Bryant L.K., Cascio C.J. (2015). Interoceptive ability and body awareness in autism spectrum disorder. J. Exp. Child Psychol..

[bib0895] Schulz A., Strelzyk F., de Sá D.S.F., Naumann E., Vögele C., Schächinger H. (2013). Cortisol rapidly affects amplitudes of heartbeat-evoked brain potentials—implications for the contribution of stress to an altered perception of physical sensations?. Psychoneuroendocrinology.

[bib0900] Seth A.K. (2013). Interoceptive inference, emotion, and the embodied self. Trends Cogn. Sci..

[bib0905] Shah P., Hall R., Catmur C., Bird G. (2016). Alexithymia, not autism: is associated with impaired interoception. Cortex.

[bib0910] Shao S., Shen K., Wilder-Smith E.P., Li X. (2011). Effect of pain perception on the heartbeat evoked potential. Clin. Neurophysiol..

[bib0915] Silani G., Bird G., Brindley R., Singer T., Frith C., Frith U. (2008). Levels of emotional awareness and autism: an fMRI study. Soc. Neurosci..

[bib0920] Silver A.J. (1990). Aging and risks for dehydration. Cleve. Clin. J. Med..

[bib0925] Sokol-Hessner P., Hartley C.A., Hamilton J.R., Phelps E.A. (2015). Interoceptive ability predicts aversion to losses. Cogn. Emot..

[bib0930] St-Onge M.P. (2005). Relationship between body composition changes and changes in physical function and metabolic risk factors in aging. Curr. Opin. Clin. Nutr. Metab. Care.

[bib0935] Steinberg L. (2007). Risk taking in adolescence new perspectives from brain and behavioral science. Curr. Direct. Psychol. Sci..

[bib0940] Stephani C., Vaca G.F.B., Maciunas R., Koubeissi M., Lüders H.O. (2011). Functional neuroanatomy of the insular lobe. Brain Struct. Funct..

[bib0945] Steptoe A., Vögele C. (1992). Individual differences in the perception of bodily sensations: the role of trait anxiety and coping style. Behav. Res. Ther..

[bib0950] Stern E.R. (2014). Neural circuitry of interoception: new insights into anxiety and obsessive-compulsive disorders. Curr. Treat. Opt. Psychiatry.

[bib0955] Stevens J.C., Cruz L.A., Hoffman J.M., Patterson M.Q. (1995). Taste sensitivity and aging: high incidence of decline revealed by repeated threshold measures. Chem. Senses.

[bib0960] Suzuki A., Akiyama H. (2013). Cognitive aging explains age-related differences in face-based recognition of basic emotions except for anger and disgust. Aging Neuropsychol. Cogn..

[bib0965] Suzuki K., Garfinkel S.N., Critchley H.D., Seth A.K. (2013). Multisensory integration across exteroceptive and interoceptive domains modulates self-experience in the rubber-hand illusion. Neuropsychologia.

[bib0970] Taylor G.J., Parker J.D., Bagby R.M., Acklin M.W. (1992). Alexithymia and somatic complaints in psychiatric out-patients. J. Psychosom. Res..

[bib0975] Teicher M.H., Anderson C.M., Ohashi K., Polcari A. (2014). Childhood maltreatment: altered network centrality of cingulate, precuneus, temporal pole and insula. Biol. Psychiatry.

[bib0980] Terasawa Y., Moriguchi Y., Tochizawa S., Umeda S. (2014). Interoceptive sensitivity predicts sensitivity to the emotions of others. Cogn. Emot..

[bib0985] Thomas L.A., De Bellis M.D., Graham R., LaBar K.S. (2007). Development of emotional facial recognition in late childhood and adolescence. Dev. Sci..

[bib0990] Tolmunen T., Heliste M., Lehto S.M., Hintikka J., Honkalampi K., Kauhanen J. (2011). Stability of alexithymia in the general population: an 11-year follow-up. Compr. Psychiatry.

[bib0995] Topsever P., Filiz T.M., Salman S., Sengul A., Sarac E., Topalli R., Gorpelioglu S., Yilmaz T. (2006). Alexithymia in diabetes mellitus. Scott. Med. J..

[bib1000] Trevisan D.A., Bowering M., Birmingham E. (2016). Alexithymia, but not autism spectrum disorder, may be related to the production of emotional facial expressions. Mol. Autism.

[bib1005] Umetani K., Singer D.H., McCraty R., Atkinson M. (1998). Twenty-four hour time domain heart rate variability and heart rate: relations to age and gender over nine decades. J. Am. Coll. Cardiol..

[bib1010] Ushiroyama T., Sugimoto O. (1993). Clinical observation of personality characteristics of perimenopausal women with undefined complaints. Ocean. J. Obst. Gynaecol..

[bib1015] Van der Does A.W., Antony M.M., Ehlers A., Barsky A.J. (2000). Heartbeat perception in panic disorder: a reanalysis. Behav. Res. Ther..

[bib1020] Van der Graaff J., Branje S., De Wied M., Hawk S., Van Lier P., Meeus W. (2014). Perspective taking and empathic concern in adolescence: gender differences in developmental changes. Dev. Psychol..

[bib1025] Van't Wout M., Aleman A., Bermond B., Kahn R.S. (2007). No words for feelings: alexithymia in schizophrenia patients and first-degree relatives. Compr. Psychiatry.

[bib1030] Verdejo-Garcia A., Clark L., Dunn B.D. (2012). The role of interoception in addiction: a critical review. Neurosci. Biobehav. Rev..

[bib1035] Waikar S.S., Mount D.B., Curhan G.C. (2009). Mortality after hospitalization with mild, moderate, and severe hyponatremia. Am. J. Med..

[bib1040] Waldstein S.R., Kauhanen J., Neumann S.A., Katzel L.I. (2002). Alexithymia and cardiovascular risk in older adults: psychosocial, psychophysiological, and biomedical correlates. Psychol. Health.

[bib1045] Warren J.L., Bacon W.E., Harris T., McBean A.M., Foley D., Phillips C. (1994). The burden and outcomes associated with dehydration among US elderly, 1991. Am. J. Public Health.

[bib1050] Weinstock H., Berman S., Cates W. (2004). Sexually transmitted diseases among American youth: incidence and prevalence estimates, 2000. Perspect. Sex Reprod. Health.

[bib1055] Werner N.S., Jung K., Duschek S., Schandry R. (2009). Enhanced cardiac perception is associated with benefits in decision-making. Psychophysiology.

[bib1060] West J.T., Horning S.M., Klebe K.J., Foster S.M., Cornwell R.E., Perrett D., Burt M., Davis H.P. (2012). Age effects on emotion recognition in facial displays: from 20–89 years of age. Exp. Aging Res..

[bib1065] Whitbourne S.K. (2012). The Aging Body: Physiological Changes and Psychological Consequences.

[bib1070] Whitehead W.E., Drescher V.M. (1980). Perception of gastric contractions and self‐control of gastric motility. Psychophysiology.

[bib1075] Whitehead W.E., Drescher V.M., Heiman P., Blackwell B. (1977). Relation of heart-rate control to heartbeat perception. Biofeedback Self-Regul..

[bib1080] Wiens S., Mezzacappa E.S., Katkin E.S. (2000). Heartbeat detection and the experience of emotions. Cogn. Emot..

[bib1085] Wildman H.E., Jones G.E. (1982). Consistency of heartbeat discrimination scores on the Whitehead procedure in knowledge-of-results—trained and untrained subjects. Psychophysiology.

[bib1090] Williams L.M., Mathersul D., Palmer D.M., Gur R.C., Gur R.E., Gordon E. (2009). Explicit identification and implicit recognition of facial emotions: I. Age effects in males and females across 10 decades. J. Clin. Exp. Neuropsychol..

[bib1095] Yashin A.I., Akushevich I.V., Arbeev K.G., Akushevich L., Ukraintseva S.V., Kulminski A. (2006). Insights on aging and exceptional longevity from longitudinal data: novel findings from the Framingham Heart Study. Age.

[bib1100] Zimmermann G. (2006). Delinquency in male adolescents: the role of alexithymia and family structure. J. Adolesc..

[bib1105] Zvolensky M.J., Smits J.A. (2007). Anxiety in Health Behaviors and Physical Illness.

